# Plasma MicroRNA Levels Differ between Endurance and Strength Athletes

**DOI:** 10.1371/journal.pone.0122107

**Published:** 2015-04-16

**Authors:** Sophie L. Wardle, Mark E. S. Bailey, Audrius Kilikevicius, Dalia Malkova, Richard H. Wilson, Tomas Venckunas, Colin N. Moran

**Affiliations:** 1 Health and Exercise Sciences Research Group, University of Stirling, Stirling, Scotland; 2 School of Life Sciences, College of Medical, Veterinary and Life Sciences, University of Glasgow, Glasgow, Scotland; 3 Lithuanian Sports University, Kaunas, Lithuania; 4 School of Medicine, College of Medical, Veterinary and Life Sciences, University of Glasgow, Glasgow, Scotland; University of Massachusetts Medical, UNITED STATES

## Abstract

**Aim:**

MicroRNAs (miRNAs) are stable in the circulation and are likely to function in inter-organ communication during a variety of metabolic responses that involve changes in gene expression, including exercise training. However, it is unknown whether differences in circulating-miRNA (c-miRNA) levels are characteristic of training modality.

**Methods:**

We investigated whether levels of candidate c-miRNAs differ between elite male athletes of two different training modalities (n = 10 per group) - endurance (END) and strength (STR) - and between these groups and untrained controls (CON; n = 10). Fasted, non-exercised, morning plasma samples were analysed for 14 c-miRNAs (miR-1, miR-16-2, miR-20a-1, miR-21, miR-93, miR-103a, miR-133a, miR-146a, miR-192, miR-206, miR-221, miR-222, miR-451, miR-499). Moreover, we investigated whether c-miRNA levels were associated with quantitative performance-related phenotypes within and between groups.

**Results:**

miR-222 was present at different levels in the three participant groups (p = 0.028) with the highest levels being observed in END and the lowest in STR. A number of other c-miRNAs were present at higher levels in END than in STR (relative to STR, ± 1 SEM; miR-222: 1.94 fold (1.73-2.18), p = 0.011; miR-21: 1.56 fold (1.39-1.74), p = 0.013; miR-146a: 1.50 fold (1.38-1.64), p = 0.019; miR-221: 1.51 fold (1.34-1.70), p = 0.026). Regression analyses revealed several associations between candidate c-miRNA levels and strength-related performance measures before and after adjustment for muscle or fat mass, but not following adjustment for group.

**Conclusion:**

Certain c-miRNAs (miR-222, miR-21, miR-146a and miR-221) differ between endurance- and resistance-trained athletes and thus have potential as useful biomarkers of exercise training and / or play a role in exercise mode-specific training adaptations. However, levels of these c-miRNAs are probably unrelated to muscle bulk or fat reserves.

## Introduction

The health benefits of regular exercise are well recognised [1]. However, the physiological adaptations that occur in response to exercise training differ according to genetic predisposition [2] and exercise modality [3,4]. These differential responses are driven by a variety of mechanisms including changes in gene expression and protein abundance [5], yet the specific mechanisms governing these molecular modifications remain poorly understood.

Recent research has centred on epigenetic control mechanisms, including microRNAs (miRNAs), and their role in regulating gene expression. miRNAs are small, non-coding ribonucleic acids (RNAs) that play a vital role in regulation of gene expression. A major part of their role is in post-transcriptional regulation through direct binding to target messenger RNAs (mRNAs), and it is estimated that at least 30% of the human gene complement is regulated by miRNAs [6]. Each miRNA binds to multiple mRNAs, and each mRNA is regulated by multiple miRNAs, providing a highly sensitive reciprocal level of regulation [7].

Differences in skeletal muscle phenotype have been associated with differences in miRNA expression levels and with genetic differences in miRNA binding sites. In Texel sheep, regulation of skeletal muscle hypertrophy has been associated with variation in a region of the *Myostatin* gene containing binding sites for muscle-specific miRNAs [8]. In humans, levels of miR-378 in skeletal muscle were significantly correlated with lean muscle mass gains following a 12 week resistance exercise training intervention [9]. In addition, levels of several other miRNAs in skeletal muscle have been shown to change following acute endurance exercise and following endurance exercise training interventions [10]. Thus, miRNAs are associated with skeletal muscle adaptation to exercise training and in combination with underlying genetic differences, may be involved in the differential phenotypic response to resistance and endurance type exercise.

miRNAs are found not only in tissues (*e*.*g*. skeletal muscle) but also in a variety of body fluids including plasma [11,12]. Outside the cell, RNases act to rapidly degrade RNA [13]; however, miRNAs are thought to be stable within the circulation primarily through incorporation into secreted exosomes, but also through co-localisation with the RNA-induced silencing complex (RISC) or via binding to high-density lipoproteins (HDL) [14]. Additionally, miRNAs appear to be actively secreted into the circulation since exosomes contain only a subset of miRNAs, present in proportions different to those in their cell type of origin [15]. Recent studies have suggested that circulating miRNAs (c-miRNAs) are involved in cell-cell communication and thus may have regulatory roles in normal physiology and in disease mechanisms [14]. c-miRNAs are remarkably stable in the circulation, easy to sample, and can be readily extracted and measured using standard qRT-PCR techniques [11]. For these reasons, the study of c-miRNAs may yield useful insight into the mechanisms of adaptation to exercise.

The mechanisms by which exercise adaptation is effected and coordinated across multiple organ systems remain somewhat unclear. Training is known to alter the health and function of not only the exercised skeletal muscle but also a number of other organs resulting in a whole body adaptive response [16]. c-miRNAs may be acting as the orchestrators of this multi-site response. Changes in c-miRNA levels have been demonstrated following a programme of endurance training [17,18] and in response to an acute endurance [17–19] or resistance [20] exercise bout. Post-exercise changes in c-miRNA levels also have been shown to differ between concentric and eccentric endurance exercise modalities [21]. Thus, the capacity for miRNAs to respond to the exercised and trained state, along with differential c-miRNA responses to exercise of differing modality, renders c-miRNAs potential biomarkers of specific exercise responses.

We aimed to examine whether there are differences in c-miRNA profiles between males involved in different long-term training regimes, and to determine whether c-miRNAs are specific markers of particular training modes. Such differences may form part of the basis for differences in physiological adaptation between individuals using different training modes. First, we investigated whether levels of candidate c-miRNAs differed between individuals at two ends of the training specificity spectrum, elite endurance (END) and strength (STR) athletes, and in comparison to age-matched, untrained controls (CON). In addition, since endurance and strength athletes differ phenotypically to such an extent, we conducted secondary analysis comparing c-miRNA levels in the two athlete groups alone. Finally, we investigated the association between c-miRNA levels and quantitative physiological variables indicative of strength or endurance training. We hypothesised that levels of the selected c-miRNAs, which have previously been shown to be exercise-responsive, would differ between the two athlete populations and also that c-miRNA levels would associate with respective quantitative variables.

## Materials and Methods

### Ethics statement

Independent ethical approval was obtained from the Lithuanian Bioethics Committee and all procedures were in accordance with the Declaration of Helsinki (2008). All participants gave full written informed consent prior to study commencement.

### Participant characteristics

Participant characteristics are summarised in Table 1. Participants were selected from a larger cohort of male athletes and controls recruited to a genetic association study (GeLA cohort) conducted at the Lithuanian Sports University and for whom plasma samples were available. The study sample comprised strength athletes (STR; n = 10), endurance athletes (END; n = 10) and age-matched non-exercising controls (CON; n = 10). Athlete participants were selected from a larger pool after phenotyping for athletic performance. The 10 strength athletes with the best performance across several strength / power tests (isokinetic peak torque of arms and legs, handgrip strength and counter-movement jump height) were selected. For the endurance subgroup, the 10 age-matched athletes with the largest relative maximal oxygen uptake (treadmill) were selected (for values see Table 1; for methods see S1 Methods). All athletes trained for 13 h per week on average, and competed regionally, nationally or internationally. Endurance athletes were involved in sports such as distance running and orienteering (sports that mostly require use of the legs), whereas strength athletes were involved in sports such as weightlifting and combat sports (which require a more equal contribution of arm and leg muscles). Concentric and eccentric exercise components were likely similar between training types. Control participants were age-matched to the athlete groups but otherwise selected randomly from the control group in the genetic association study, and did not compete in any competitive sport, or partake in any organised physical activity on more than two occasions per week.

**Table 1:** Participant characteristics by group.

	CON	STR	END
n	10	10	10
Age (years)	24.0 ± 2.8	22.2 ± 2.1	22.6 ± 3.7
Body mass (kg)	79.3 ± 15.6	84.8 ± 10.2	70.6 ± 7.2 †
Muscle mass (MM; kg)	41.8 ± 7.4	50.7 ± 6.7 *	42.8 ± 4.3 †
Fat mass (FM; kg)	24.3 ± 9.6	19.7 ± 4.2	13.9 ± 1.2 * †
BMI (kg·m^-2^)	24.4 ± 3.1	25.6 ± 2.6	22.2 ± 2.5 †
VO_2_ max (mL·kg^-1^·min^-1^)	43.5 ± 2.4	50.4 ± 6.5 *	66.9 ± 4.7 * †
VO_2_ max (mL·kg^-0.75^·min^-1^)	129.2 ± 7.0	152.8 ± 5.8*	189.1 ± 6.3 * †
Wingate fatigue index (W·s^-1^)	6.5 ± 1.2	6.1 ± 0.7	4.8 ± 1.5 * †
Isokinetic knee extension peak torque (Nm; 30°·s^-1^)	507.4 ± 122.9	605.6 ± 121.5	459.3 ± 54.2 †
Isokinetic elbow extension peak torque (Nm; 30°·s^-1^)	115.4 ± 22.3	152.6 ± 46.5 *	94.2 ± 12.9 * †
Handgrip strength (kg)	121.4 ± 11.9	148.2 ± 14.6 *	111.7 ± 14.3 * †
Counter-movement jump height (cm)	36.1 ± 5.8	42.6 ± 6.3 *	37.7 ± 5.5

All values are mean ± SD. With the exception of age, all measures are significantly different by ANOVA (p < 0.05).

* significantly different from CON by t-test;

^†^ significantly different from STR by t-test (p < 0.05). VO_2_ max (mL·kg^-0.75^·min^-1^) has been included for comparison to other studies.

### Sampling

Participants visited the laboratory in a fasted state and at least 12 h post-exercise. Venous blood samples (10 mL) were collected from all study participants in EDTA vacutainers and centrifuged at 3000 rpm for 15 min at room temperature for plasma separation. Plasma was immediately aliquoted into 1.5 mL microcentrifuge tubes for storage at -80°C pending subsequent analysis.

On the other visits to the laboratories over the period of several days to 3 weeks, a large variety of phenotype measurements were collected. These measurements included anthropometric characteristics, tests of handgrip strength, isokinetic dynamometry, pull-ups, vertical jumps, an agility shuttle run, a 30 m sprint, a Wingate test, and a continuous ramp-up spiroergometric treadmill test to exhaustion. Details of the testing procedures and protocols are provided in S1 Methods. Fat mass (FM) was calculated from the sum of 4 skinfold measurements (triceps, biceps, subscapular and suprailiac) using the body fat percentage calculation of Durnin & Womersley [22]. Muscle mass (MM) was estimated using standardised height and skinfold-corrected girth measurements of the forearm, calf and thigh within the equation of Martin *et al*. [23].

### RNA extraction and complementary DNA (cDNA) synthesis

All molecular and statistical analyses were conducted at the University of Stirling. RNA was extracted from plasma samples using the miRNeasy Mini Kit (Qiagen Ltd., West Sussex, UK) according to the manufacturer’s instructions with a starting volume of 200 μL. From the resulting RNA eluate, cDNA was synthesised using the miScript II RT Kit (Qiagen Ltd., West Sussex, UK) according to the manufacturer’s instructions using 12 μl template RNA. cDNA reactions were diluted from 20 μL to 250 μL with double-distilled water and loaded into deep well plates for storage at -20°C until further analysis.

### miRNA analysis

Commercially available quantitative polymerase chain reaction (qRT-PCR) primer assays (Qiagen Ltd., West Sussex, UK) were used for miRNA analysis (target miRNAs: miR-1, miR-20a-1, miR-21, miR-133a, miR-146a, miR-206, miR-221, miR-222, miR-499; control miRNAs: miR-16-2, miR-93, miR-103a, miR-192, miR-451). The candidate c-miRNAs measured in the present study were selected due to their observed upregulation at rest in response to endurance rowing training [17], for their status as myomiRs [24] or because we initially believed them to be suitable for use as control genes [25]. miRNA levels were measured in triplicate, using the synthesised cDNA, with the miScript SYBR Green PCR kit (Qiagen Ltd., West Sussex, UK). A standard curve (1 in 10 dilution series starting with a 1 in 10 dilution, using a pooled sample consisting of cDNA from all participants in all three groups) was included on each plate to assess whether all assays were in the linear range. The final 15 μL reaction volume comprised: 2x SYBR Green mix (7.5 μL), 10x miScript universal primer (1.5 μL), 10x miScript primer assay (1.5 μL) and cDNA dilution (4.5 μL).

Real-time quantitative polymerase chain reactions (qRT-PCR) were run on the Quantica real-time thermal cycler system (Techne, Bibby Scientific Ltd., Staffordshire, UK). An initial activation step (95°C; 15 min) preceded 40 cycles of denaturation (94°C; 15 s), annealing (55°C; 30 s) and elongation / extension (70°C; 30 s). Reactions were completed with a melting curve for quality control to ensure that only a single amplicon was present in each reaction. All cycle threshold (Ct) values were within the linear range of the standard curve. Ct outliers were removed using the median absolute deviation method (also known as the modified Z-score; [26]) with the maximum acceptable threshold set at 3.5. Control samples (pooled cDNA from each participant) were included on each plate to allow standardisation of the Ct values for experiments that spanned several plates.

Five control assays were selected (see above for miRNA controls list) based on previous reports showing expression stability within plasma [25]. Normfinder software [27] was used to determine which single gene or group of genes was at the most stable level within the samples. miR-93 alone was found to be most stable across the three groups and was therefore used to determine the relative expression of the target genes, calculated using the 2^-ΔΔCt^ method [28]. Consistent with miR-93 being the most stable miRNA between groups, data from other reports indicate that the other 4 miRNAs may not have been good candidates for control genes due to their association with other stimuli including cancer [29]. Thus, these 4 miRNAs were added to the set analysed as candidate genes.

### miRNA target prediction

Pathways targeted by our candidate miRNAs were predicted using the web-based tool, miRSystem [30]. miRSystem integrates several databases (Kegg, Biocarta, Pathway Interaction Database, Reactome and GO molecular function) to enable prediction of gene targets and targeted pathways.

### Statistical analysis

#### Power calculations

Power calculations for the study design were conducted using G*Power3 [31] based on means and standard deviations of relative c-miRNA levels reported by Baggish *et al*. [17]. Power calculations were also performed on a number of physiological measures from the current study to ensure sample sizes were adequate for detecting between-group differences in these physiological variables. An n of 10 offered 80% power to detect between-group differences in mean miRNA levels of 0.60 SDs by ANOVA, α = 0.05. The smallest significant between-group difference reported by Baggish *et al*. [17] was 1.33 SDs. In all analyses described below, we have adopted the principle that because there is prior evidence for associations between levels of the c-miRNAs tested and performance-related phenotypes, an α of *p* = 0.05 is appropriate for each test.

#### c-miRNA analysis by participant group

Statistical analysis was conducted using Minitab software (version 16; Minitab, State College, PA). Box-Cox transformations were applied to non-normally distributed data and normal distributions were confirmed using the Ryan-Joiner test. All statistical tests were conducted on normally distributed data. Between-group differences in miRNA levels for STR, CON and END subgroups were determined by one-way ANOVA. In the case of statistical significance by ANOVA, Tukey’s *post-hoc* tests were implemented. Direct comparisons of miRNA levels between the two athlete groups only were conducted by means of Student’s t-tests.

#### Association of c-miRNA expression with performance-related phenotypes

Regression analyses (GLM) were conducted between c-miRNA levels and quantitative phenotypes related to strength or endurance exercise. Following initial regression analyses between levels of each candidate c-miRNA and each phenotypic measure, we ran models incorporating group (*i*.*e*. STR, CON, END) and tissue mass (fat mass (FM) and muscle mass (MM)) as predictive variables. Adjusting for tissue mass is important as some c-miRNA levels are correlated with tissue mass measures, and cellular miRNA pools in muscle and adipose tissue could be influencing circulating levels; this potentially confounding factor therefore needs to be accounted for. Adjusting for group further accounts for unmeasured intrinsic differences between the different types of athletes that could also be confounding uncorrected associations. All values are expressed as mean ± standard error of the mean (SEM) unless stated otherwise.

## Results

### c-miRNA analysis by group

**Fig 1:**
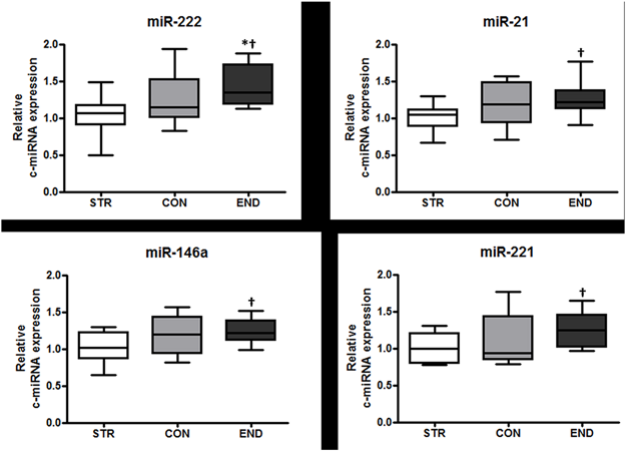
Relative expression levels of miR-222 (A), -21 (B), -146a (C), and -221 (D) in STR, CON and END. Box plots depict the range (upper and lower whiskers), median (centre line) and interquartile range (edge of boxes). * significantly different between all groups (One way ANOVA; p < 0.05); † significantly different from STR (t-test; p < 0.05).

Circulating levels of 14 miRNAs were measured in the athlete and control groups. Table 2 shows mean relative levels for each group for each of the test miRNAs, standardised to the reference gene (miR-93) and to levels in the control (CON) group. One-way ANOVA revealed a statistically significant difference (p = 0.028; Fig 1A) in miR-222 levels between the three groups, with levels nominally being higher than CON in the endurance (END) group and lower than CON in the strength (STR) group. The *post-hoc* Tukey’s test revealed a significant difference between STR and END (p = 0.011).

**Table 2:** Levels of all measured c-miRNAs.

c-miRNA	CON			STR			END			ANOVA p value
	Mean	95% CI	SEM	Mean	95% CI	SEM	Mean	95% CI	SEM	
**miR-1**	1	0.32–3.11	0.65–2.08	0.84	0.42–1.67	0.63–1.26	1.17	0.33–4.18	0.74–2.71	0.910
**miR-16-2**	1	0.51–1.96	0.75–1.49	1.3	0.55–3.07	0.92–2.20	0.76	0.30–1.93	0.53–1.36	0.675
**miR-20a-1**	1	0.44–2.27	0.71–1.65	1.03	0.43–2.42	0.72–1.74	0.62	0.25–1.50	0.43–1.07	0.657
**miR-21**	1	0.73–1.37	0.86–1.19	0.77	0.61–0.96	0.69–0.87	1.20 †	0.96–1.48	1.08–1.34	0.074
**miR-103a**	1	0.74–1.35	0.87–1.18	0.8	0.62–1.03	0.71–0.92	1.14	0.82–1.59	0.98–1.37	0.251
**miR-133a**	UD	-	-	UD	-	-	UD	-	-	-
**miR-146a**	1	0.76–1.32	0.88–1.16	0.75	0.57–0.97	0.66–0.86	1.12 †	0.95–1.32	1.03–1.22	0.069
**miR-192**	1	0.65–1.53	0.82–1.27	1.15	0.65–2.03	0.89–1.60	0.88	0.38–2.05	0.62–1.48	0.845
**miR-206**	1	0.11–9.18	0.55–5.17	1.54	0.48–4.95	1.00–3.28	0.28	0.05–1.52	0.16–0.91	0.392
**miR-221**	1	0.71–1.41	0.85–1.21	0.9	0.71–1.14	0.80–1.02	1.36 †	1.08–1.71	1.22–1.54	0.119
**miR-222**	1	0.73–1.37	0.86–1.19	0.66	0.44–1.00	0.55–0.83	1.29 †	1.03–1.62	1.16–1.46	0.028 *
**miR-451**	1	0.57–1.74	0.78–1.38	1.34	0.77–2.33	1.05–1.85	0.70	0.38–1.29	0.54–1.00	0.307
**miR-499**	1	0.47–2.13	0.73–1.58	0.53	0.25–1.11	0.39–0.83	0.47	0.13–1.68	0.30–1.09	0.485

Mean values are expressed relative to CON and all measurements have been standardised relative to levels of miR-93 as a control. UD = Undetectable.

* significantly different between the three groups (One way ANOVA; p < 0.05);

^†^ significantly different from STR by t-test (p < 0.05).

Plasma levels of all other c-miRNAs were not significantly different between groups by ANOVA (p > 0.05; Table 2). However, as we expected to find differences between elite athletes undergoing very different training regimes, we also conducted t-tests comparing the athlete groups only. This secondary analysis revealed higher plasma levels of three other miRNAs in END than in STR: miR-21 (p = 0.013), miR-146a (p = 0.019) and miR-221 (p = 0.026) (Fig 1B–1D), with CON intermediate in mean level, as found for miR-222.

### Association of c-miRNA expression with performance-related phenotypes

The differences in circulating levels of a subset of the miRNAs tested could reflect a response of various aspects of cellular physiology to different training regimes. Thus, we investigated whether levels of the four miRNAs showing differences between the athlete groups were correlated with values for performance in a range of tests that are indicative of the physiological adaptation to different training regimes. We investigated these correlations with and without adjustment for athlete group and anthropometric measures (FM and MM) in the regression models. These corrections enabled us to account for inherent training / phenotypic differences between the groups and to control for differences in body composition between groups. Differences in muscle bulk or fat reserves may influence levels of c-miRNAs either via rates of production or via rates of uptake by these tissues, both of which have an important influence on capacity for, or the adaptation to, exercise. The characteristics of participants in each group for these measures are summarised in S1 Table and results of the regression models are summarised in S2–S5 Tables.

Plasma miR-222 levels (S2 Table) were positively correlated with height, body mass, muscle mass and fat mass after adjustment for group (p < 0.05). Also, plasma miR-222 levels were positively correlated with a strength-related performance measure, isokinetic leg flexion peak torque at various contraction velocities, after adjustment for group (p = 0.018 to 0.043), but not in the basic model, nor after adjustment for MM or FM. None of the other performance measures were correlated with levels of this c-miRNA.

Plasma miR-21 levels (S3 Table) were not associated with anthropometric parameters, but were negatively correlated with a subset of strength / power and endurance-related measures in some models. These strength-related associations were true for handgrip strength in the unadjusted model (p = 0.039) and for some velocities of isokinetic leg and arm extension peak torque in the unadjusted and MM-adjusted models (p ranges from 0.015 to 0.044). A similar negative correlation was seen with an anaerobic endurance parameter, the Wingate fatigue index, in the unadjusted and MM-adjusted models (p < 0.045).

Plasma miR-146a levels (S4 Table) were positively correlated with height in the group-adjusted (p = 0.023), MM-adjusted (p < 0.001) and FM-adjusted (p = 0.016) models and negatively correlated with BMI in the MM-adjusted (p = 0.010) and FM-adjusted (p = 0.005) models. Levels of miR-146a were not associated with endurance- or power-related measurements, but were negatively correlated with a subset of strength-related measures in some models. These strength-related associations were true for handgrip strength in the unadjusted, and both MM- and FM-adjusted, models (p < 0.027), and for most measures of isokinetic arm flexion and extension peak torque in the unadjusted, and both MM- and FM-adjusted, models (p ranges from 0.002 to 0.045). No significant associations (other than for height) were observed following adjustment for group.

Plasma miR-221 levels (S5 Table) were positively associated with height after MM- or FM-adjustment (p < 0.038) and negatively correlated with BMI after adjustment for MM (p = 0.016). Levels of this c-miRNA were not associated with endurance- or power-related measurements, but were negatively associated with some strength-related measures. These strength-related negative associations included handgrip strength in the unadjusted and FM-adjusted models (p < 0.024) and particularly in the MM-adjusted model (p = 0.001). Moreover, most measures of isokinetic arm flexion and extension peak torque in the MM-adjusted models (p ranges from 0.008 to 0.046) also were negatively associated with plasma miR-221 levels. Again, no significant associations were observed following adjustment for group.

It should be noted that a number of myomiRs (miR-1, -133a, -206, -499) were assessed in the present investigation; however, they are found at very low levels in the circulation, which affected measurement consistency in our analysis. Such measurement inconsistencies also have been noted in some reports [18,32] in the context of c-miRNAs and we would recommend a pre-amplification step when investigating these miRNAs in future.

### Predicted miRNA targets

Given the differences between the athlete groups in a subset of the miRNAs tested (miR-21, miR-146a, miR-221 and miR-222) and the association between levels of these c-miRNAs and a subset of performance parameters, we conducted an analysis to ascertain whether common mRNA targets were shared to a significant extent by these c-miRNAs. Predictions using miRSystem indicated that these four miRNAs, when added into the model simultaneously, target mRNAs contained in a total of 736 pathways across all integrated databases (data not shown). The top 8 pathways targeted by all four miRNAs together (arbitrarily selected based on a score > 3.0) are shown in Table 3.

**Table 3:** miRSystem pathway analysis.

Database	Pathway	Total union targets (of total genes in the pathway)	Score
Pathway interaction database	Direct p53 effectors	31 (of 137)	4.430
Pathway interaction database	PDGFR-β signalling	26 (of 126)	3.862
Pathway interaction database	c-myb transcription factor network	17 (of 81)	3.511
Go molecular function (Tier 2)	Protein binding transcription factor activity	45 (of 369)	3.445
Kegg	TGF-β signalling	17 (of 84)	3.347
Kegg	Pathways in cancer	43 (of 325)	3.310
Kegg	MAPK signalling	36 (of 272)	3.167
Pathway interaction database	SHP2 signalling	14 (of 54)	3.143

Union targets refers to genes targeted by all 4 miRNAs; score is deduced from the sum of the weight of its miRNA multiplied by its enrichment, minus the log (p value) from the predicted gene targets. The weight for one miRNA is calculated by dividing its absolute expression value by the absolute sum of the expression values for all input miRNAs [30].

## Discussion

We report a significant difference in circulating miR-222 levels between male endurance athletes, strength athletes and age-matched untrained controls. Moreover, in endurance athletes, plasma levels of miR-21, miR-146a and miR-221, as well as miR-222, were significantly higher than in strength athletes. Levels of these c-miRNAs were significantly associated with performance parameters indicative of training mode, such as isokinetic peak torque and VO_2_max, particularly in tissue-corrected models. To the best of our knowledge this study is the first to investigate levels of c-miRNAs in distinct athlete groups and in comparison to untrained controls in a single study, and also is the first to offer a comprehensive range of quantitative phenotypic variables for comparative assessment in the same individuals.

Data from previous investigations of the candidate c-miRNAs and their direction of change in association with exercise adaptation have been somewhat inconsistent. Moreover, the potential for differential regulation by endurance and resistance exercise has not previously been addressed in a single study. In response to both an acute, single-bout endurance exercise test and a 12 wk period of longitudinal endurance exercise training, Baggish *et al*. [17] reported an increase of the same four c-miRNAs found to be higher in endurance athletes here (miR-21, -146a, -221 and -222). In another study, plasma miR-146a and -221 levels were reduced 3 days after performing an acute bout of resistance exercise [20], consistent with our findings in strength trained athletes, although longitudinal data to relate acute effects to sustained training responses are lacking. These data suggest that levels of particular c-miRNAs may change in opposite directions following endurance *vs*. resistance exercise. However, other studies have reported conflicting results. Some studies report no change in circulating levels of miR-21 and -222 following acute resistance exercise [20]; others report lower circulating levels of miR-21, and -222 in individuals with high (151.2 mL·kg^-0.75^·min^-1^) compared to low (104.2 mL·kg^-0.75^·min^-1^) VO_2_max [33]; or a reduction in miR-21 in previously trained males after 12 wk of further endurance exercise training [18]. However, our endurance athletes have an even higher VO_2_max (see Table 1) and may not be directly comparable to these aforementioned studies. These contrasting findings may suggest an increased responsiveness of these c-miRNAs to uncontrolled environmental influences and consideration should be given to subtle differences in subgroup selection, study design and participant physiology.

Athletes training and competing in diverse exercise modes display clear phenotypic differences. Endurance athletes are typically lighter, leaner, have higher cardiorespiratory fitness, and are less strong and less powerful than their resistance-trained counterparts. These differences are underlain by genetic predisposition and training mode, and these influences combine and interact in complicated ways, being mediated largely through epigenetic regulation. By investigating associations between levels of our differentially regulated c-miRNAs and quantitative phenotypic variables we can better understand this epigenetic regulation of phenotype. A strength of our study was that we took the important additional step of adjusting for the type of athlete / training mode in our statistical analyses, which has been done only rarely in previous studies. We found that correcting for group had the clearest influence on associations between levels of our candidate c-miRNAs and physiological characteristics. This finding may indicate that the unmeasured intrinsic differences (or other unmeasured environmental differences, such as subtle differences in diet) between different kinds of athlete, or athletes on different training regimes, have a role in driving the uncorrected associations and justify the adjustment for group. Furthermore, we also adjusted for two measures of tissue mass, which, again, has been done only rarely in previous studies. It can be hypothesised that tissue mass may correlate with the pool size of c-miRNAs available for endocytosis / exocytosis. However, the fact that we observed associations in the models corrected for tissue mass suggests that between-group differences in c-miRNA levels are likely unrelated to tissue mass, and hence does not provide support for the ‘pool’ hypothesis. At this stage it is possible only to speculate about alternative explanations, but possible factors explaining the between-group differences include training characteristics, innate contractile properties of the muscle, and habitual dietary differences. Further work is required to better define the relationship between changes in c-miRNA levels and performance / health-related phenotypes.

Aerobic capacity is a hallmark of the endurance trained state. In the present study, the endurance-trained cohort had significantly greater relative VO_2_max than the strength-trained cohort. However, VO_2_max was not significantly associated with levels of any of the candidate miRNAs, in either the uncorrected or group- / tissue mass-corrected models. We did note that miR-21 was significantly inversely associated with Wingate fatigue index, a measure derived from the Wingate test of anaerobic endurance capacity [34], in the uncorrected (r = -0.39) and muscle mass-corrected (r = -0.38) models, but the other mi-RNAs showed no association with this measure. Thus, with the potential exception of miR-21, differences in c-miRNA levels between groups are not involved in differences in aerobic or anaerobic endurance capacity between, or within, groups. This conclusion is therefore consistent with findings from Bye *et al*.[33] who reported a lack of association between VO_2_max and miR-222 and only a weak association (r = -0.20) between endurance capacity (VO_2_max) and miR-21.

Strength and power phenotypes are characteristic of resistance-trained individuals. Thus, whether markers of strength and / or power performance (which were significantly higher in our strength-trained cohort compared to the endurance-trained athletes) were significantly associated with c-miRNA levels in uncorrected and group- / tissue mass-corrected models was investigated. In the uncorrected model, total handgrip strength was significantly negatively associated with circulating levels of miR-21, -146a and -221. We had expected these associations to exist in the absence of any corrections because of the selection criteria we applied for inclusion in each group. However, upon correction for group, these associations are no longer apparent; suggesting that the variation in c-miRNA levels within groups does not associate with the variation in total handgrip strength within groups. In this regard, candidate c-miRNA levels do not associate with strength *per se*; rather c-miRNA levels must associate with other between-group differences that are explanatory for the variation. Differences in fat mass may explain some of the variation in plasma miR-21 levels with regards to strength phenotypes, but neither fat mass nor muscle mass explains the variation in levels of any other c-miRNA. In fact, correction for group appears to be the only correction factor that alters the associations relative to the uncorrected model for all differentially regulated c-miRNAs. For miR-21, -146a and-221, negative associations with several markers of strength, most notably isokinetic peak torque at various contraction velocities, are apparent in the uncorrected and / or muscle mass-corrected models. For miR-21 and -221 in particular, it is likely that individuals with lower levels of these c-miRNAs are stronger per unit of muscle mass (*i*.*e*. possess greater specific force). The only c-miRNA to differ significantly between all groups (including controls), miR-222, is not significantly associated with any parameter in the uncorrected or tissue mass-corrected models. With group accounted for, miR-222 significantly associates with some anthropometric and strength measures. However, the association between miR-222 and isokinetic peak torque at faster flexion velocities is actually positive in nature. This direction of association is in contrast to previously reported associations and to what we would expect from the case-control analysis. These findings suggest that, rather than training mode, other between-group factors; for example, dietary habits, physical size and skeletal muscle fibre type differences, may be having a greater effect on miR-222 level. Although the lack of clear associations between miR-222 and performance phenotypes is disappointing following the results from the case-control analyses, this disconnect highlights the value of including quantitative phenotype measures in study designs. Since miRNAs have inhibitory actions on gene expression, the lower levels of candidate c-miRNAs observed in our strength cohort would suggest removal of suppression of gene expression. Considering the miRSystem pathway analysis demonstrated that the associated set of miRNAs target genes were involved in skeletal muscle remodelling, our conclusion would be that remodelling processes governed by the products of these genes may be involved in strength training-related muscle adaptation.

Although no tissue was collected for gene expression analysis of our c-miRNA targets, bioinformatic analyses with miRSystem [30] at least allows us to predict the mRNA targets of the candidate miRNAs investigated in our study and consider whether these are consistent with the observed phenotypic differences between groups. Predicted pathways targeted by our differentially regulated miRNAs included signalling pathways involved in cell growth and inflammation. Specifically, platelet-derived growth factor receptor (PDGF-R), transforming growth factor beta (TGF-β) and mitogen-activated protein kinase (MAPK) signalling pathways as well as transcription factor networks (c-Myb transcription factor network, protein binding transcription factor activity) were identified. PDGF-R signalling has been implicated in blood vessel growth via recruitment of vascular smooth muscle cells [35] and appears to be regulated by miR-146a in particular [36]. TGF-β also is involved in cell growth and control of inflammation [37] while MAPK signalling occurs in several tissues and is implicated in many diverse physiological processes including gene expression regulation and cell differentiation [38]. In the context of exercise training, well-known adaptations include enhanced angiogenesis leading to a greater microvascular volume [39], anti-inflammatory responses leading to a more optimal hormonal environment [16] and enhanced skeletal muscle cell differentiation and proliferation [40]. Thus, it is logical that these pathways may be involved in the training response although exact mechanisms of action cannot be determined from this analysis.

Despite the limited influence of correction for muscle mass or fat mass within our models, given that c-miRNAs must be released by a tissue and ultimately act on mRNAs within a tissue, it is important to consider how c-miRNAs relate to tissue mass. Within an exercise context, an obvious source of non-specific miRNA release into plasma is from muscle-damaging exercise. Banzet *et al*. [21] revealed enhanced levels of muscle-specific c-miRNAs (myomiRs; miR-1, -133a, -133b and -208) following a bout of eccentric exercise, compared to pre-exercise levels (with no change observed following concentric endurance exercise). This led the authors to conclude that changes in c-miRNA levels were driven by the damaging nature of eccentric exercise and muscle leakage in the post-exercise period (up to 6 h). However, markers of muscle damage were not different between groups in that study, not all muscle miRNAs are present in the circulation and the direction of change of c-miRNA levels is not always consistent with non-specific release from tissue (*i*.*e*. increased levels in the circulation) [21,41]. Furthermore, recent reports [18,19] suggest limited associations between c-miRNA levels and muscle damage, or a failure to detect muscle-specific miRNAs following exhaustive exercise. Therefore, we believe it is unlikely that muscle leakage is the primary cause of changes in c-miRNA levels, although more work is required to confirm this belief, and to better understand other aspects of c-miRNA regulation in general.

In summary, a subset of c-miRNAs (miR-21, -146a, -221 and -222) are associated with training-related performance parameters in a manner consistent with them being involved in the whole-body adaptive response to differential forms of exercise training. Thus, these c-miRNAs may be useful biomarkers of exercise training and / or have potential roles in exercise mode-specific training adaptations. However, their levels in the circulation appear to be unrelated to having more muscle bulk or larger fat reserves (with the exception of the miR-21 relationship with differences in fat mass), and thus it is unlikely that they are limited by the amount of tissue available for the release / uptake of c-miRNAs. Nonetheless, whether the presence of c-miRNAs in the circulatory system is regulated primarily by tissue exocytosis or endocytosis still remains of interest and further research is warranted to investigate the relationship between observed differences in c-miRNA levels and target gene expression in relevant tissues within the context of exercise training.

## Supporting Information

S1 Fig Relative miRNA expression of targets (A), myomiRs (B) and control miRNAs (C) for each of the 3 groups (STR, CON, END). Bars are means ± 95% confidence limits; * significantly different between all groups (One way ANOVA; p < 0.05); † significantly different from STR (t-test; p < 0.05). miR-133a was not consistently detected and thus is omitted from the myomiRs plot.
(TIF)

S1 Methods Organisation and methods of the study. (DOC)

S1 Table Participant characteristics of all performance-related variables. Values are mean ± standard error of the mean (SEM). * significantly different from CON; † significantly different from STR (p < 0.05).
(DOCX)

S2 Table Regression analyses of miR-222 and performance-related variables before and after correction for group, MM or FM. Shaded boxes denote p < 0.05. Coefficients represent the direction and magnitude of the response. Regression analyses were performed on z-score data.
(DOCX)

S3 Table Regression analyses of miR-21 and performance-related variables before and after correction for group, MM or FM. Shaded boxes denote p < 0.05. Coefficients represent the direction and magnitude of the response. Regression analyses were performed on z-score data.
(DOCX)

S4 Table Regression analyses of miR-146a and performance-related variables before and after correction for group, MM or FM. Shaded boxes denote p < 0.05. Coefficients represent the direction and magnitude of the response. Regression analyses were performed on z-score data.
(DOCX)

S5 Table Regression analyses of miR-221 and performance-related variables before and after correction for group, MM or FM. Shaded boxes denote p < 0.05. Coefficients represent the direction and magnitude of the response. Regression analyses were performed on z-score data.
(DOCX)
