# Supplementary figures and images for: Plasma MicroRNA Levels Differ between Endurance and Strength Athletes
Source: PLoS One. 2015 Apr 16;10(4):e0122107. doi: 10.1371/journal.pone.0122107 (PMC4400105; doi:10.1371/journal.pone.0122107)

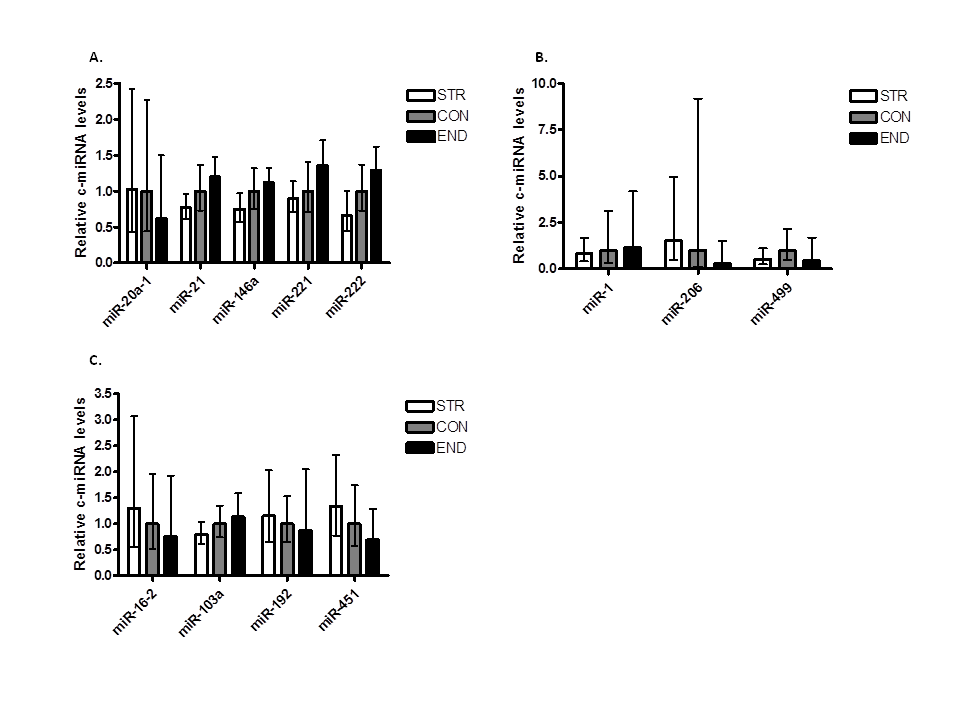

Supplement: S1 Fig — Bars are means ± 95% confidence limits; * significantly different between all groups (One way ANOVA; p < 0.05); † significantly different from STR (t-test; p < 0.05). miR-133a was not consistently detected and thus is omitted from the myomiRs plot. (TIF) [file pone.0122107.s001.tif]
