# Supplementary material for: Plasma MicroRNA Levels Differ between Endurance and Strength Athletes
Source: PLoS One. 2015 Apr 16;10(4):e0122107. doi: 10.1371/journal.pone.0122107 (PMC4400105; doi:10.1371/journal.pone.0122107)

**S Methods. Organisation and methods of the study**

**Organisation of the study**

Participants undertook a standard 3-day test battery. Before each of the performance tests they were instructed on how to perform the test properly for consistency between participants. Tests were performed and measurements taken during three visits to the laboratories in the order presented below. There were 3–10 days of rest between visits for non-athletes, and 1–10 days for athletes (athletes were allowed to have only one day rest between the visits to the lab because of their training commitments and because the tests were deemed to be not as demanding for them as for untrained participants). In between the visits, all participants completed a log of their training history (training experience in years and level of achievements), as well as training frequency (in sessions/week) and volume (in hours/week) of training – both averaged over the preceding 4 weeks.

**Visit 1**

**Anthropometrics**

Body mass was measured on electronic scales (Tanita TBF-300, Japan). A Harpenden anthropometer was used to measure stature, sitting height and leg length (from the iliac crest). Body mass index (BMI) was calculated as the ratio of body mass (kg) to squared body height (m^2^). A measuring tape was used to measure girths of the forearm, calf and thigh, and callipers were used to measure thickness of skinfolds.

**Handgrip strength**

General upper body strength was tested with the frequently used handgrip test (4). A Hydraulic Hand Dynamometer (Model J00105, Lafayette Instrument Company, Lafayette, IN) was used to measure grip strength. Participants were seated at a table with their knees and elbow of the tested arm flexed to 90°. The dynamometer was adjusted to the individual hand size, and before each attempt participants used a towel to dry their palms to ensure good grip. On each attempt, participants were required to squeeze the dynamometer with maximal efforts for 2 seconds. Three attempts with 30 seconds of rest in between were allowed, and the best result was recorded for each hand.

# Following the tests outlined above, participants performed a warm-up consisting of 6–8 min stationary cycling (Ergoselect 100, Ergoline GmbH, Bitz, Germany) at ~70 rpm with the power (Watts) adjusted to roughly equivalent to the participant’s body mass in kg, followed by some light stretching exercises.

**Isokinetic dynamometry**

Knee and elbow flexion and extension peak torques were measured by isokinetic dynamometry (Biodex Pro3, USA) at 30, 90 and 180 °/s angular speeds. Each set consisted of 3 repetitions of consecutive flexion/extensions cycles, and each repetition was performed with maximal efforts through all the range of motion. Sets always started with 30 °/s angular speed, and were followed 90 and 180 °/s angular speeds. Each of the sets was separated by 90 seconds of passive rest, and subjects started with their right leg, then left leg, right arm and left arm.

**Pull-ups**

A standard rigid gymnastic bar (2.5 cm diameter) at a height of 2.6 m was used for the pull up test. Participants were instructed to give their maximum efforts to perform as many chin-ups as possible at their preferred speed but without passively hanging on the bar. One trial was allowed, and care was taken the test was performed properly (2).

**Visit 2**

The tests on Visit 2 were preceded by the same warm-up as on Visit 1.

**Counter movement jump (CMJ) and Squat jump (SJ)**

For the CMJ, the participant was required to squat quickly to a 90° angle at the knee and immediately jump up as high as he could. For the SJ, participants remained in the squat (90° knee angle) for 3–5 seconds before propelling upwards maximally without any additional drop in the centre of gravity. CMJ attempts preceded SJ attempts. During jumps of both modes, arms were held akimbo and the knee angles as well as the vertical modes of the jumps were inspected visually by the same experienced researcher. A rest period of at least 30 s between jumps was chosen to minimise metabolic perturbations that could affect muscle function. Jumps were performed on a portable multicomponent force platform (Kistler, type 9286AA, Switzerland), which measured the vertical component of ground reaction force at a frequency of 1000 Hz. Software (Kistler BioWare with Performance module (version 3.06c)) was used to collect and analyse force data during the jumps. The height (H) of the jump was calculated using the following formula: H (cm) = 122.625 * (T_f_)^2^, where T_f_ is flight time in seconds (1). After practicing for 2 trials on each jump mode, three attempts of each mode were allowed, and the highest jump of each mode was recorded for subsequent analysis.

**Agility (10 x 5 m) shuttle run**

Bipedal dexterity and leg power/agility were evaluated using a 10 x 5-m (5 laps) maximal shuttle run on a smooth hard surface following the protocol of Christou et al. (3). After a submaximal trial for one lap with 1-minute rest, a maximum test (one trial) was performed. The time of 5 laps was taken using a hand-held stopwatch.

**30 m sprint run**

The sprint run was performed on a smooth, hard surface. Four fixed field (without reflectors) electronic photocells (NewTest Powertimer; Oulu, Finland) were positioned at distances of 0 m, 10 m, 20 m and 30 m and at a height of 1 m. The time for three consecutive 10 m sprints was recorded to an accuracy of 0.001 seconds. Three trials with a 2-3 minute recovery in between were performed. The fastest time of the three attempts was recorded for subsequent analysis.

**Wingate anaerobic cycling test**

When maximal pedal revolution rate was attained on a mechanical cycle ergometer (Monark 824E; Sweden) in the absence of any resistance, the participant gave a signal and a brake weight equalling 7.5 percent (approximated to the nearest 0.1 kg) of the participant’s own body mass was applied to initiate the 30 seconds all-out test. Pedalling cadence was recorded by means of custom made software (AdRec, Vilnius, Lithuania) using an accelerometer mounted inside the sock of the participant. The number of revolutions of each 5-s interval was used to obtain peak power and 30-s average power using standard formulas. Wingate Fatigue Index (in percent) was calculated as 100% minus the percent power difference between the first and the last of 5-s intervals.

**Visit 3 (**
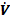
**O_2_ max test)**

To measure
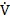
O_2_ max, a ramp treadmill protocol of continuous incremental running til volitional exhaustion was used. The warm-up before the test consisted of 5 min of jogging on the treadmill at a comfortable speed, followed by some light stretching exercises. The participant started the test by jogging at 7 km/h for 3 minutes at the initial gradient of 1%, and then the speed of the treadmill belt increased by 0.1 km/h each 6 seconds. The treadmill speed remained constant when it reached 20 km/h, and then the gradient of the treadmill was ramp increased at ramp by an average of 1% every minute from the initial 1%. Throughout the test, breath-by-breath gas analysis was performed using an Oxycon Mobile gas analyser (Viasys, Germany), and heart rate (HR) was recorded with a HR monitor (810s; Polar, Finland). During the test, participants were verbally encouraged to attain their maximal efforts.
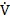
O_2_ max (in L·min^-1^) was calculated as the highest average over 20 consecutive seconds, and then relative
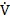
O_2_ was calculated by indexing it per kg of body mass and per kg^-0.75^ of body mass.

**Supplemental references**

1. Bosco C, Viitasalo JT, Komi P V, Luhtanen P. Combined effect of elastic energy and myoelectrical potentiation during stretch-shortening cycle exercise. *Acta physiologica Scandinavica* 1982;114(4):557–65.

2. Burnstein BD, Steele RJ, Shrier I. Reliability of fitness tests using methods and time periods common in sport and occupational management. *Journal of athletic training* 2011;46(5):505–13.

3. Christou M, Smilios I, Sotiropoulos K, Volaklis K, Pilianidis T, Tokmakidis SP. Effects of resistance training on the physical capacities of adolescent soccer players. *Journal of strength and conditioning research / National Strength & Conditioning Association* 2006;20(4):783–91.

4. Kamimura T, Ikuta Y. Evaluation of grip strength with a sustained maximal isometric contraction for 6 and 10 seconds. *Journal of rehabilitation medicine* 2001;33(5):225–9.

**S1 Table**. Participant characteristics of all performance-related variables. Values are mean ± standard error of the mean (SEM). * significantly different from CON; † significantly different from STR (p < 0.05).

|  |  | **CON** | **STR** | **END** |
| --- | --- | --- | --- | --- |
| **Anthropometric-related** | Height (cm) | 179.7 ± 2.5 | 181.5 ± 1.9 | 178.4 ± 2.0 |
|  | Body mass (kg) | 79.3 ± 4.9 | 84.6 ± 3.6 | 70.6 ± 2.3 |
|  | BMI (kg·m^-2^) | 24.4 ± 1.0 | 25.6 ± 0.8 | 22.2 ± 0.8 |
| **Power-related** | No. of pull ups | 6.0 ± 1.1 | 14.3 ± 1.2 * | 9.5 ± 1.4 † |
|  | CMJ height (cm) | 36.1 ± 1.8 | 42.6 ± 2.0 * | 37.7 ± 1.7 |
|  | SJ height (cm) | 29.1 ± 1.4 | 35.3 ± 2.0 * | 29.8 ± 1.6 † |
|  | Shuttle run (s) | 20.7 ± 0.5 | 20.3 ± 0.5 | 19.5 ± 0.5 |
|  | Wingate 1st 5s (revs) | 11.9 ± 0.3 | 12.9 ± 0.2 * | 12.7 ± 0.5 |
|  | Sprint 0-10 (s) | 2.0 ± 0.0 | 1.9 ± 0.0 * | 1.8 ± 0.0 * |
|  | Sprint total (s) | 4.7 ± 0.1 | 4.4 ± 0.0 * | 4.4 ± 0.0 * |
| **Strength-related** | Handgrip total (kg) | 121.4 ± 3.7 | 148.2 ± 4.6 * | 111.7 ± 4.5 † |
|  | IKleg ext 30 °·s^-1^ (Nm) | 507.4 ± 38.9 | 605.6 ± 38.4 | 459.3 ± 17.1 † |
|  | IKleg flex 30 °·s^-1^ (Nm) | 256.5 ± 16.5 | 308.4 ± 20.9 | 253.8 ± 13.5 |
|  | IKleg ext 90 °·s^-1^ (Nm) | 420.3 ± 28.0 | 496.4 ± 32.1 | 408.1 ± 12.7 † |
|  | IKleg flex 90 °·s^-1^ (Nm) | 226.5 ± 17.1 | 279.8 ± 18.0 * | 237.7 ± 9.8 |
|  | IKleg ext 180 °·s^-1^ (Nm) | 334.1 ± 24.4 | 383.2 ± 20.5 | 318.1 ± 9.4 † |
|  | IKleg flex 180 °·s^-1^ (Nm) | 197.2 ± 13.3 | 232.7 ± 14.0 | 205.2 ± 7.7 |
|  | IKarm ext 30 °·s^-1^ (Nm) | 115.4 ± 7.0 | 152.6 ± 14.7 * | 94.2 ± 4.1 *† |
|  | IKarm flex 30 °·s^-1^ (Nm) | 125.8 ± 6.1 | 167.9 ± 13.0 * | 113.1 ± 4.7 † |
|  | IKarm ext 90 °·s^-1^ (Nm) | 102.0 ± 6.2 | 135.2 ± 15.1 * | 81.9 ± 5.3 *† |
|  | IKarm flex 90 °·s^-1^ (Nm) | 115.5 ± 6.2 | 149.0 ± 13.5 * | 96.6 ± 4.2 *† |
|  | IKarm ext 180 °·s^-1^ (Nm) | 89.4 ± 5.6 | 118.0 ± 12.0 * | 72.4 ± 3.6 *† |
|  | IKarm flex 180 °·s^-1^ (Nm) | 99.6 ± 5.4 | 124.6 ± 11.7 | 86.3 ± 3.6 † |
| **Endurance-related** | Wingate total (revs per 30 s) | 48.5 ± 1.3 | 56.6 ± 0.8 * | 59.9 ± 1.7 * |
|  | Wingate fatigue index | 6.5 ± 0.4 | 6.1 ± 0.2 | 4.8 ± 0.5 *† |
|  | 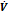O_2_ max (L·min^-1^) | 3.4 ± 0.2 | 4.2 ± 0.1 * | 4.6 ± 0.1 * |
|  | 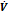O_2_ max (mL·kg^-1^·min^-1^) | 43.5 ± 0.7 | 50.4 ± 2.1 * | 66.9 ± 1.5 *† |

**S2 Table.** Regression analyses of miR-222 and performance-related variables before and after correction for group, MM or FM. Shaded boxes denote p < 0.05. Coefficients represent the direction and magnitude of the response. Regression analyses were performed on z-score data.

**S3 Table**. Regression analyses of miR-21 and performance-related variables before and after correction for group, MM or FM. Shaded boxes denote p < 0.05. Coefficients represent the direction and magnitude of the response. Regression analyses were performed on z-score data.

**S4 Table**. Regression analyses of miR-146a and performance-related variables before and after correction for group, MM or FM. Shaded boxes denote p < 0.05. Coefficients represent the direction and magnitude of the response. Regression analyses were performed on z-score data.

**S5 Table.** Regression analyses of miR-221 and performance-related variables before and after correction for group, MM or FM. Shaded boxes denote p < 0.05. Coefficients represent the direction and magnitude of the response. Regression analyses were performed on z-score data.

**S1 Figure**. Relative miRNA expression of targets (A), myomiRs (B) and control miRNAs (C) for each of the 3 groups (STR, CON, END). Bars are means ± 95 % confidence limits; * significantly different between all groups (One way ANOVA; p < 0.05); † significantly different from STR (t-test; p < 0.05). miR-133a was not consistently detected and thus is omitted from the myomiRs plot.


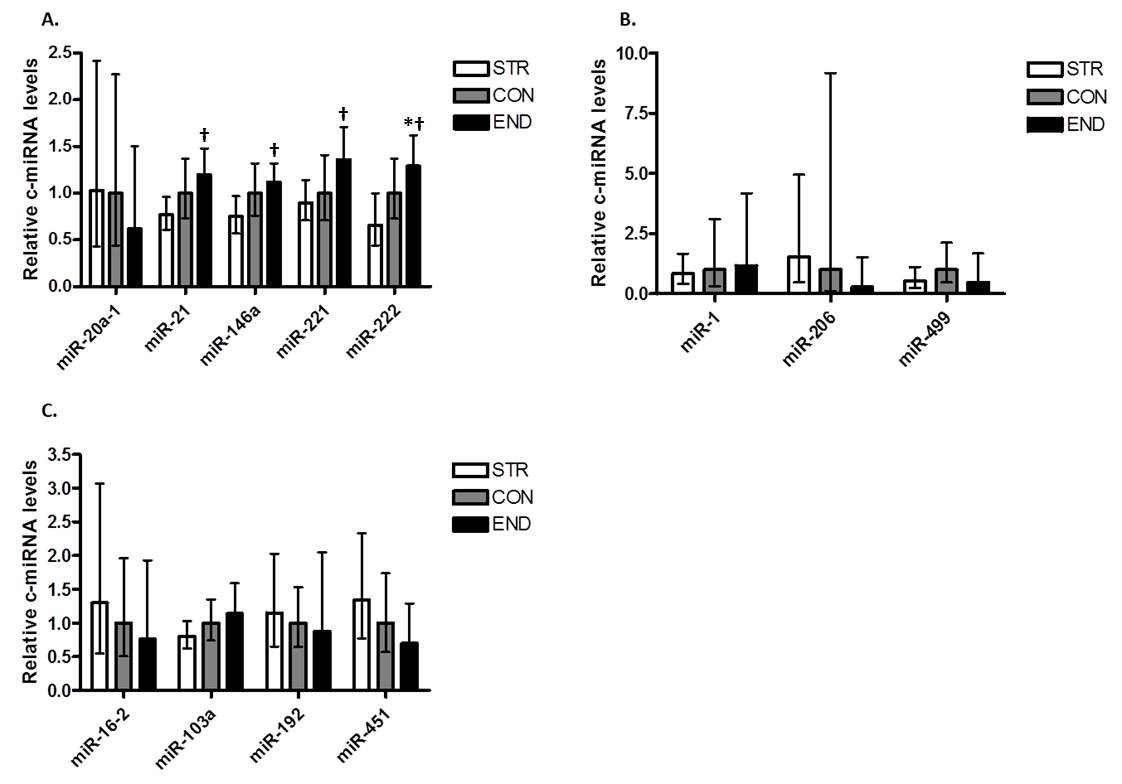

Supplement: S1 Methods — (DOC) [file pone.0122107.s002.doc]
