# Supplementary material for: Plasma MicroRNA Levels Differ between Endurance and Strength Athletes
Source: PLoS One. 2015 Apr 16;10(4):e0122107. doi: 10.1371/journal.pone.0122107 (PMC4400105; doi:10.1371/journal.pone.0122107)
Supplement: S1 Table — Values are mean ± standard error of the mean (SEM). * significantly different from CON; † significantly different from STR (p < 0.05). (DOCX) [file pone.0122107.s003.docx]

**Supporting Information Table 1**. Participant characteristics of all performance-related variables.

|  |  | **CON** | **STR** | **END** |
| --- | --- | --- | --- | --- |
| **Anthropometric-related** | Height (cm) | 179.7 ± 2.5 | 181.5 ± 1.9 | 178.4 ± 2.0 |
|  | Body mass (kg) | 79.3 ± 4.9 | 84.6 ± 3.6 | 70.6 ± 2.3 |
|  | BMI (kg·m^-2^) | 24.4 ± 1.0 | 25.6 ± 0.8 | 22.2 ± 0.8 |
| **Power-related** | No. of pull ups | 6.0 ± 1.1 | 14.3 ± 1.2 * | 9.5 ± 1.4 † |
|  | CMJ height (cm) | 36.1 ± 1.8 | 42.6 ± 2.0 * | 37.7 ± 1.7 |
|  | SJ height (cm) | 29.1 ± 1.4 | 35.3 ± 2.0 * | 29.8 ± 1.6 † |
|  | Shuttle run (s) | 20.7 ± 0.5 | 20.3 ± 0.5 | 19.5 ± 0.5 |
|  | Wingate 1st 5s (revs) | 11.9 ± 0.3 | 12.9 ± 0.2 * | 12.7 ± 0.5 |
|  | Sprint 0-10 (s) | 2.0 ± 0.0 | 1.9 ± 0.0 * | 1.8 ± 0.0 * |
|  | Sprint total (s) | 4.7 ± 0.1 | 4.4 ± 0.0 * | 4.4 ± 0.0 * |
| **Strength-related** | Handgrip total (kg) | 121.4 ± 3.7 | 148.2 ± 4.6 * | 111.7 ± 4.5 † |
|  | IKleg ext 30 °·s^-1^ (Nm) | 507.4 ± 38.9 | 605.6 ± 38.4 | 459.3 ± 17.1 † |
|  | IKleg flex 30 °·s^-1^ (Nm) | 256.5 ± 16.5 | 308.4 ± 20.9 | 253.8 ± 13.5 |
|  | IKleg ext 90 °·s^-1^ (Nm) | 420.3 ± 28.0 | 496.4 ± 32.1 | 408.1 ± 12.7 † |
|  | IKleg flex 90 °·s^-1^ (Nm) | 226.5 ± 17.1 | 279.8 ± 18.0 * | 237.7 ± 9.8 |
|  | IKleg ext 180 °·s^-1^ (Nm) | 334.1 ± 24.4 | 383.2 ± 20.5 | 318.1 ± 9.4 † |
|  | IKleg flex 180 °·s^-1^ (Nm) | 197.2 ± 13.3 | 232.7 ± 14.0 | 205.2 ± 7.7 |
|  | IKarm ext 30 °·s^-1^ (Nm) | 115.4 ± 7.0 | 152.6 ± 14.7 * | 94.2 ± 4.1 *† |
|  | IKarm flex 30 °·s^-1^ (Nm) | 125.8 ± 6.1 | 167.9 ± 13.0 * | 113.1 ± 4.7 † |
|  | IKarm ext 90 °·s^-1^ (Nm) | 102.0 ± 6.2 | 135.2 ± 15.1 * | 81.9 ± 5.3 *† |
|  | IKarm flex 90 °·s^-1^ (Nm) | 115.5 ± 6.2 | 149.0 ± 13.5 * | 96.6 ± 4.2 *† |
|  | IKarm ext 180 °·s^-1^ (Nm) | 89.4 ± 5.6 | 118.0 ± 12.0 * | 72.4 ± 3.6 *† |
|  | IKarm flex 180 °·s^-1^ (Nm) | 99.6 ± 5.4 | 124.6 ± 11.7 | 86.3 ± 3.6 † |
| **Endurance-related** | Wingate total (revs per 30 s) | 48.5 ± 1.3 | 56.6 ± 0.8 * | 59.9 ± 1.7 * |
|  | Wingate fatigue index | 6.5 ± 0.4 | 6.1 ± 0.2 | 4.8 ± 0.5 *† |
|  | $\dot{\boldsymbol{V}}$O_2_ max (L·min^-1^) | 3.4 ± 0.2 | 4.2 ± 0.1 * | 4.6 ± 0.1 * |
|  | $\dot{\boldsymbol{V}}$O_2_ max (mL·kg^-1^·min^-1^) | 43.5 ± 0.7 | 50.4 ± 2.1 * | 66.9 ± 1.5 *† |

Values are mean ± standard error of the mean (SEM). * significantly different from CON; † significantly different from STR (p < 0.05).
