# Supplementary material for: Plasma MicroRNA Levels Differ between Endurance and Strength Athletes
Source: PLoS One. 2015 Apr 16;10(4):e0122107. doi: 10.1371/journal.pone.0122107 (PMC4400105; doi:10.1371/journal.pone.0122107)
Supplement: S5 Table — Shaded boxes denote p < 0.05. Coefficients represent the direction and magnitude of the response. Regression analyses were performed on z-score data. (DOCX) [file pone.0122107.s007.docx]

**Supporting information Table 5.** Regression analyses of miR-221 and performance-related variables before and after correction for group, MM or FM.

Shaded boxes denote p < 0.05. Coefficients represent the direction and magnitude of the response. Regression analyses were performed on z-score data.
